# Platelet preservation in cardiac surgery using minimally invasive extracorporeal circulation versus optimized cardiopulmonary bypass

**DOI:** 10.1177/02676591221130173

**Published:** 2022-09-29

**Authors:** Brian Beairsto, Cyril Serrick, Amanda Fernandez, Myriam Lafreniere-Roula, Mitesh Badiwala, Keyvan Karkouti, Vivek Rao

**Affiliations:** 1 Department of Perfusion, Peter Munk Cardiac Centre, 7989 University Health Network, Toronto, ON, Canada; 2 Ted Rogers Computational Program, 7989 University Health Network, Toronto, ON, Canada; 3 Department of Cardiovascular Surgery, Peter Munk Cardiac Centre, 7989 University Health Network, Toronto, ON, Canada; 4 Department of Anesthesiology, 7989 University Health Network, Toronto, ON, Canada

**Keywords:** extracorporeal circulation, mini-circuit, coagulation, platelet function

## Abstract

**Background:**

Minimally invasive extracorporeal circulation (MiECC) is employed as a strategy to attenuate the physiologic disturbance caused by cardiopulmonary bypass. The aim of this study was to compare the coagulation profile of MiECC to an optimized conventional extracorporeal circuit (OpECC) with regards to platelet function, rotational thromboelastometry and blood product usage.

**Methods:**

A retrospective analysis of coronary artery bypass grafting operations using either MiECC or OpECC was performed at a single institution.

**Results:**

A total of 112 patients were included, with 61 receiving MiECC and 51 OpECC patients. OpECC patients had a significantly larger BSA (1.95+/- 0.22m2 vs 1.88 +/- 0.18m2, *p* = 0.034), than those who received MiECC. No difference between groups was observed regarding red blood cell, plasma, and platelet transfusions. Functional platelet count during the warming phase of cardiopulmonary bypass was found to be higher in the MiECC group ((136 (102–171) x10^9^/L vs 109 (94–136) x10^9^/L), *p* = 0.027), as were functional platelets as a percent of total platelet count ((86 (77–91)% vs 76 (63–82)%), *p* = 0.003). There were no significant differences between other outcomes such as operative mortality, incidence of stroke, and intensive care unit length of stay.

**Conclusion:**

While we did not see a difference in blood transfusions, MiECC resulted in a statistically significant advantage over OpECC with regards to preservation of functional platelets.

## Introduction

The development and use of the heart lung machine by John Gibbon was a revolution in cardiac surgery, allowing open heart procedures to be performed with a measure of control.^1^ Despite being used as a routine life support measure during modern cardiac surgery, cardiopulmonary bypass (CPB) continues to cause a number of derangements to the hemostatic system. Passage of blood through the CPB circuit causes contact activation, triggering both the complement system and the intrinsic pathway of the coagulation cascade.^2^ Platelets are also activated by the artificial surface of the extracorporeal circuit, resulting in reduced platelet count and impaired function following CPB. Furthermore, the need for a crystalloid priming solution causes hemodilution, thereby reducing hematocrit and oxygen delivery during CPB. These derangements can lead to coagulopathy and significant bleeding post-operatively, which has been linked to blood product usage.^3^

Advancement of extracorporeal technologies has operated with the intent to mitigate the negative consequences of CPB through decreased prime volumes and improved biocompatibility. This has led to many centers trying to optimize conventional CPB through the use of biocompatible coated circuits and oxygenators with integrated arterial filters, centrifugal pumps and shortened tubing. Furthermore, other centers have taken extra steps to improve biocompatibility by adapting a minimally invasive extracorporeal circulation (MiECC) system. MiECC combines current perfusion science and engineering to create a CPB circuit with the goal of drastically attenuating the physiologic response to CPB.^4^ A MiECC system typically employs a number of strategies to achieve this, including elimination of the venous reservoir, shortening of CPB tubing length, biocompatible coated tubing, avoidance of reinfusing unwashed cardiotomy suction blood, and routine use of a cell salvage device. Numerous studies have been conducted to study the effect of MiECC on the minimization of hemodilution and reduction of red blood cell transfusion.^5–8^ However, there are relatively fewer published papers related to the effect of MiECC on the coagulation system compared with conventional extracorporeal circulation.^9^

The primary purpose of this retrospective observational study was to compare the coagulation profiles of patients who underwent CABG using either MiECC or optimized conventional extracorporeal circulation (OpECC) and how that relates to transfusion requirements and clinical outcomes. We hypothesized that MiECC would better preserve the coagulation profile, and reduce the need for red blood cell transfusion.

## Materials and methods

The MiECC strategy was introduced at our centre in March 2019. Data was retrospectively collected on isolated CABG performed by 2 surgeons using MiECC or OpECC between March 2019 and January 2020. The use of MiECC was determined by the availability of appropriately trained perfusionists.

### Cardiopulmonary bypass

A type III MiECC circuit was used as defined by the Minimal Invasive Extracorporeal Technologies International Society.^4^ The MiECC circuit consisted of a ⅜ inch venous line, Capiox BT15 bubble trap, Medtronic Affinity centrifugal pump, Medtronic Fusion oxygenator with integrated filter, and ⅜ inch arterial line. A hard shell cardiotomy reservoir was used to collect blood returned from an aortic root vent and reinfused directly into the venous line (all products from Medtronic Inc; Minneapolis, MN). All components were heparin bonded with Cortiva bioactive surface with the exception of the bubble trap and holding cardiotomy. Suction to a cell salvage device was used before, during, and after CPB instead of cardiotomy suction. Cardiotomy suction was available, but was only activated in an emergency situation if there was unexpected bleeding.

The OpECC circuit consisted of a Balance coated (non-heparin based hydrophilic polymer coating) ½ inch venous line, ⅜ inch arterial line, Medtronic Affinity Fusion venous reservoir, Medtronic Affinity centrifugal pump, and Cortiva coated Medtronic Fusion oxygenator with integrated filter. Cell salvage was also used in OpECC, but cardiotomy suction of unwashed shed blood directed back into the venous reservioir was preferred during CPB. Other than the necessary component changes to eliminate the venous reservoir, the OpECC circuit was afforded the same strategies often reserved for MiECC (Table 1).

**Table 1.** Comparative extracorporeal circuit features.

	OpECC	MiECC
Venous reservoir	Yes	No
Centrifugal pump	Medtronic affinity - balance	Medtronic affinity - cortiva
Biocompatible tubing	Balance	Cortiva
Oxygenator	Medtronic fusion - cortiva	Medtronic fusion - cortiva
Cell saver used	Pre CPB/Post CPB	Pre CPB/CPB/Post CPB
Venous bubble trap	No	Yes
Pump suction unprocessed	Yes	No
RAP	Yes	Yes
Static prime volume	930 ml	930 ml
Dynamic prime volume	1300 ml	930 ml
ACT target	>480 s	>480 s
Temperature on CPB	34.0°C	34.0°C

Priming of both CPB circuits was accomplished using plasmaltye-A, 5000 units of heparin and 25g of mannitol. Static prime refers to the minimum volume required to prime the CPB circuit at zero flow and was calculated to be 930 ml for both systems. Dynamic prime refers to the minimum volume required to safely maintain flow through the system without entraining air. The dynamic priming volume of the OpECC circuit is 1300 ml while the dynamic priming volumes of the MiECC circuit is the same as the static prime due to it being a totally closed system. Retrograde autologous priming (RAP) was performed in both groups before initiation of CPB as long as the patient remained hemodynamically stable. Cardioplegia was delivered using the Quest MPS microplegia system in both MiECC and OpECC (Quest Medical Inc; Allen, TX). Heparin management was unchanged between groups, with a target ACT > 480 s measured using the Hemochron Signature Elite (Werfen; Bedford, MA) to initiate and maintain cardiopulmonary bypass. Protamine was dosed at a ratio of 0.7 mg of protamine to neutralize 1 mg of heparin. If the post protamine ACT remained elevated above the baseline, additional protamine was given at the discretion of the anesthesiologist. Temperature management was achieved by allowing patients to cool to 34.0°C, followed by re-warming to an oropharyngeal temperature of 37.0°C. Our transfusion trigger during and after CPB was 70 g/L and 80 g/L respectively.

Percent drop in hemoglobin after initiation, and to the end of CPB, was calculated using the first or last measured hemoglobin on CPB respectively, and the hemoglobin measured immediately pre-CPB.

### Blood sampling and analysis

Whole blood samples for rotational thromboelastometry and functional platelet counts were collected from the sampling manifold of the CPB circuit during the warming phase of CPB once the patient reached an oropharyngeal temperature of 36.0°C. Platelet counts were performed using the BC-3600 device (Helena Laboratories; Beaumont, TX). Whole blood was added in the amount of 1 ml each into two collection vials, one of which containing 1.80 mg of EDTA, and a second vial containing 10 μg liquid collagen in 3.2% sodium citrate to cause platelet activation. A platelet count was performed in each sample. Total platelet counts were derived from the sample mixed with EDTA. Non-functional platelets were derived in the collagen mixed sample as those that did not aggregate. Functional platelet count was derived by subtracting the difference.

Thromboelastometry was performed using Instrumentation Laboratory’s ROTEM system (Werfen; Bedford, MA). The EXTEM assay activates clot formation using thromboplastin, thereby allowing assessment of factors I, II, V, VII, X, platelet function, and fibrinolysis. The FIBTEM assay activates clot formation in the same way, with the addition of cytochalasin D to block platelets, allowing assessment of fibrin formation independent of platelet function. The EXTEM assay was achieved by combining 20 μl ex-tem reagent (tissue factor, phospholipids, heparin inhibitor) with 20 μl star-tem reagent (0.2 mol/l CaCl_2_ in HEPES buffer (pH 7.4), and 0.1% sodium azide) and 300 μl citrated whole blood. The FIBTEM assay was achieved by combining 20 μl ex-tem reagent (tissue factor, phospholipids, heparin inhibitor) with 20 μl fib-tem reagent (cytochasalin D solution, 0.2 mol/l CaCl_2_ in HEPES buffer (pH 7.4)) and 300 μl of whole blood.

Preoperative standard lab tests (fibrinogen, INR, aPTT, platelet count on a CBC) were drawn within a week of the scheduled surgery and accessed retrospectively. Standard coagulation tests were drawn intraoperatively after weaning from CPB.

### Statistical analysis

Statistical analysis was performed using the R software program. Baseline clinical and surgical characteristics as well as blood coagulation results and perioperative complications and outcomes were summarized using descriptive statistics. Continuous variables were summarized in terms of median and interquartile range (IQR), or mean with standard deviation. Dichotomous and polytomous variables were summarized in terms of frequencies and proportions.

## Results

Fifty-one CABG cases were performed in the study period with OpECC. Preoperative patient characteristics are summarized in Table 2. Minimally invasive extracorporeal circulation was used to perform isolated coronary bypass procedures in 60 patients and a single isolated aortic valve replacement during the study period (Table 3). Preoperative patient characteristics were similar between the two groups. However, there was a significant difference in the size of the patients as represented in the patients’ body surface area (BSA) with OpECC patients being significantly larger than the MiECC patients (1.95+/- 0.22m2 vs 1.88 +/- 0.18m2, *p* = 0.034).

**Table 2.** Preoperative patient characteristics.

	OpECC (*n* = 51)	MiECC (*n* = 61)	P
Age (mean ± SD.)	64.1 ± 10.2	66.3 ± 8.9	0.24
Male gender (%)	45 (88)	49 (80)	0.31
Weight (mean (SD), kg)	82.9 ± 16.7	77.3 ± 13.2	0.05
BSA (mean (SD), m^2^)	1.95 (0.22)	1.88 (0.18)	**0.034**
Diabetes (%)	28 (55)	31 (51)	0.71
Hypertension (%)	43 (84)	53 (87)	0.79
Dialysis (%)	1 (2)	2 (3)	>0.99
Cerebrovascular disease (%)	4 (8)	4 (7)	>0.99
Ejection fraction (median (IQR), %)	56.0 (43–60.0)	58.0 (52.0–60.0)	0.32
Atrial fibrillation (%)	2 (4)	4 (7)	0.69

**Table 3.** Intraoperative CPB characteristics.

	OpECC (n = 51)	MiECC (n = 61)	P
Procedure (%)			0.61
AVR, PFO closure	0 (0)	1 (2)	
CABG + LV aneurysm repair	2 (4)	0 (0)	
CABGx1	1 (2)	0 (0)	
CABGx2	7 (14)	7 (11)	
CABGx3	19 (37)	26 (43)	
CABGx4	16 (31)	22 (36)	
CABGx5	5 (10)	5 (8)	
CABGx6	1 (2)	0 (0)	
CPB time (median (IQR), minutes)	60 (48–73)	61 (53–76)	0.25
Cross clamp time (median (IQR), minutes)	50 (36–60)	49 (42–62)	0.41
CPB prime (median (IQR), ml)	535 (464–1035)	380 (330–480)	**<0.001**
CPB retrograde autologous prime (mean (SD), ml)	382 ± 245	545 ± 134	**<0.001**
CPB fluid balance (median (IQR), ml)	988 (674–1588)	751 (408–1252)	0.06
Hgb pre CPB (median (IQR), g/L)	128 (123–140)	124 (111–136)	0.06
First hgb on CPB (median (IQR), g/L)	103 (94–112)	102 (88–115)	0.97
Hgb % drop CPB initiation (median (IQR), %)	21 (16–25)	18 (14–23)	**0.04**
Last hgb on CPB (median (IQR), g/L)	101 (90–113)	104 (91–115)	0.70
Hgb % drop CPB end (median (IQR), %)	20 (15–24)	15 (12–21)	**0.002**
Cell salvage blood loss from surgical field (median (IQR), ml)	350 (147–589)	500 (300–850)	**0.008**
Processed cell salvage RBCs returned to patient (mean (SD), ml)	166 ± 198	466 ± 244	**<0.001**

**Hgb % drop CPB initiation** = percentage drop of hemoglobin from Hgb pre CPB to First Hgb on CPB. **Hgb % drop CPB end** = percentage drop of hemoglobin from Hgb pre CPB to Last Hgb on CPB.

**Figure 1.**
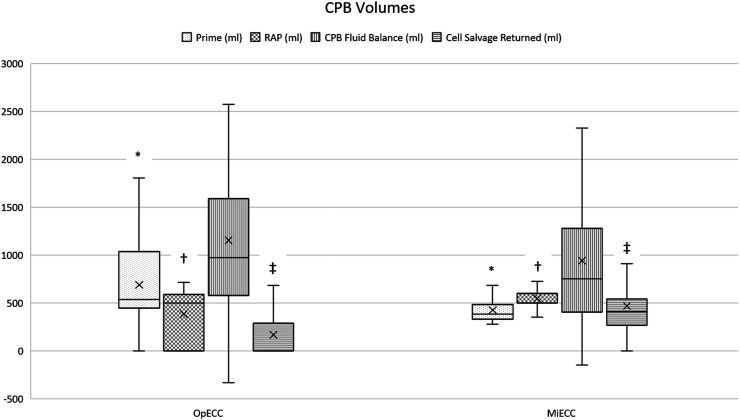
Comparative volume administration. *P<0.001 †P<0.001 ‡P<0.001. CPB = cardiopulmonary bypass. RAP = retrograde autologous prime. CPB = cardiopulmonary bypass.

Intraoperatively, neither CPB time (60 min (48–73) vs 61 min (53–76), *p* = 0.25) nor cross clamp time (50 min (36–60) vs 49 min (42–62), *p* = 0.41) were different between groups. Priming volume of the CPB circuit was lower when MiECC was used (535 ml (464–1035) vs 380 ml (330–480), p = <0.001) (Table 3). Retrograde autologous priming (RAP) was performed more frequently on patients in the MiECC group (*n* = 59) compared to OpECC (*n* = 38), with a larger amount of volume being removed when MiECC was used (600 ml (550–600) vs 500 ml (485–590), p = <0.001) (Figure 1).

**Figure 2.**
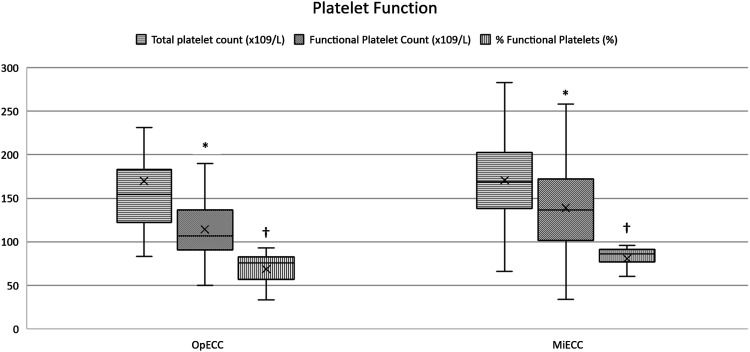
Platelet count and function derived from aggregometry. *P=0.027 †P=0.003. Samples were drawn when 36.0°C was reached during warming phase of cardiopulmonary bypass.

Intraoperative coagulation markers are summarized in Table 4. No statistically significant difference in preoperative platelet count (235 +/-99 x10^9^/L vs 233 +/- 79 x10^9^/L) or total platelet count during the rewarming phase of CPB (157 (126–182) x10^9^/L vs 168 (138–201) x10^9^/L) was observed. We did, however, observe a statistically higher number of functional platelets when MiECC was used (136 (102–171) x10^9^/L) compared to OpECC (109 (94–136) x10^9^/L). Furthermore, the percentage of functional platelets was significantly higher in the MiECC group (86 (77–91)% vs 76 (63–82)%) (Figure 2).

**Table 4.** Intraoperative coagulation profile.

	OpECC (*n* = 31)	MiECC (*n* = 57)	P
Preoperative platelet count (mean (SD), x10^9^/L)	235 (99)	233 (79)	0.89
Total platelet count (median (IQR), x10^9^)	157 (126–182)	168 (138–201)	0.23
Functional platelet count (median (IQR), x10^9^)	109 (94–136)	136 (102–171)	**0.03**
Functional platelet % (median (IQR))	76 (63–82)	86 (77–91)	**0.003**
EXTEM CT (median (IQR), seconds)	83 (69–96)	90 (81–106)	**0.006**
EXTEM CFT (median (IQR), seconds)	103 (84–126)	85 (75–107)	0.05
EXTEM MCF (median (IQR), mm)	61 (58–68)	64 (60–67)	0.49
EXTEM lysis (median (IQR), %)	0 (0–2)	1 (0–2)	0.45
Fibtem MCF (median (IQR), mm)	13 (12–17)	15 (13–18)	0.16
Fibrinogen post CPB (median (IQR), g/L)	1.93 (1.70–2.73)	1.79 (1.52–2.23)	0.23
INR post CPB (median (IQR))	1.4 (1.2–1.6)	1.5 (1.3–1.6)	0.32
aPTT (median (IQR), seconds)	28.9 (26.3–32.5)	28.6 (25.5–36.1)	0.98
CBC platelet count post CPB (median (IQR), x10^9^)	139 (108–156)	143 (97–168)	0.83

The use of blood products is summarized in Table 5. Red blood cells were transfused in 45% and 38% of patients in the OpECC and MiECC groups respectively, however, this was not statistically different (*p* = 0.45). No difference was observed for other blood products, including platelets, plasma, or prothrombin complex (Table 5). There were also no significant differences in operative mortality, stroke, or ICU stay (Table 6).

**Table 5.** Blood product usage.

	OpECC (*n* = 51)	MiECC (*n* = 61)	P
Received red blood cell transfusion (n (%))	23 (45)	23 (38)	0.45
Received plasma transfusion (n (%))	1 (2)	0 (0)	0.46
Received platelet transfusion (n (%))	5 (10)	7 (11)	>0.99
Received prothrombin complex transfusion (n (%))	0 (0)	1 (2)	>0.99

**Table 6.** Patient outcomes.

	OpECC (*n*=51)	MiECC (*n*=61)	P
Operative mortality (%)	1 (2)	0 (0)	0.46
Reoperation for bleeding (%)	0 (0)	2 (3)	0.5
Reoperation for graft occlucion (%)	0 (0)	0 (0)	—
Reoperation for non-cardiac reasons (%)	0 (0)	0 (0)	—
Sepsis (%)	1 (2)	0 (0)	0.46
Embolic stroke (%)	1 (2)	1 (2)	>0.99
Prolonged ventilation (>48h) (%)	1 (2)	1 (2)	>0.99
Renal failure (%)	0 (0)	0 (0)	—
ICU stay (median (IQR), hours)	26.5 (22.8–44.7)	24.5 (21.8–29.6)	0.27

## Discussion

This study was performed to assess differences in platelet function and coagulation parameters between OpECC and MiECC. Results from the ROTEM analysis show a prolonged clot formation time (CFT) for OpECC when compared to MiECC, however, the CFT for both groups fell within normal limits. Clotting time (CT) was slightly prolonged beyond normal limits for both groups, with MiECC showing a statistically significant longer time to reach CT than OpECC. In previous work comparing the effects on TEG, both R time and K time after 45 min of CPB were prolonged when conventional CPB was used compared to minimally invasive CPB.^10^ Rahe-Meyer and colleagues found at the end of CPB, CT was prolonged to a greater degree in their standard heart lung machine, whereas CFT was prolonged to a greater degree in their minimally invasive bypass circuit.^11^ Interestingly, this relationship is in the opposite direction found here. In the present study, it is not clear if using MiECC versus OpECC had any impact on the ROTEM profile Table 7.

**Table 7.** Abbreviations and acronyms.

Term	Abbreviation
Activated clotting time	ACT
Cardiopulmonary bypass	CPB
Conventional extracorporeal circulation	CECC
Clot formation time	CFT
Clotting time	CT
Coronary artery bypass grafting	CABG
Ethylenediaminetetraacetic acid	EDTA
Hemoglobin	Hbg
Interquartile range	IQR
Minimally invasive extracorporeal circulation	MiECC
Optimized conventional extracorporeal circulation	OpECC
Red blood cell	RBC
Research ethics board	REB
Retrograde autologous priming	RAP
Thromboelastography	TEG

Total platelet count in samples collected during the warming phase of CPB were no different between groups; however, functional platelet counts were significantly higher in the MiECC cohort. Superior preservation of functional platelets that occurred in the MiECC group is represented by the percent functionality of the total count ((86 (77–91)% vs 76 (63–82)%). Rahe-Meyer and colleagues observed a similar result using collagen activated platelet aggregation, finding superior ability for platelets to aggregate using MiECC after 30 min on, and post CPB.^10^ Of important note, this difference was no longer observed by postoperative day one. A similar pattern has been noted when examining the magnitude of platelet activation during CPB by measuring the release of beta-thromboglobulin. By the end of CPB, platelets were activated to a higher degree when conventional bypass was used compared to MiECC.^12^ One potential reason for preservation of platelet function using MiECC is that, compared to OpECC, there is an attenuation of thrombin generation during CPB.^13^ Lower levels of intraoperative thrombin generation will serve as a weaker stimulus for platelet activation and aggregation.^9^ The intrinsic pathway of the coagulation cascade is likely blunted during MiECC due to reduced tubing surface area and blood air interface, and avoiding reinfusion of shed blood directly into the CPB circuit, although it is not clear to which degree each of these individual components are responsible. In fact, when studying MiECC in the literature, determining a cause-and-effect relationship is often difficult due to a number of changes commonly applied to the MiECC group that is not offered to the conventional cohort. For example, one or more of the following are often used with MiECC but withheld for conventional extracorporeal circulation (CECC): centrifugal pumps, coated tubing, use of cell salvage, avoidance of pump suction, and retrograde autologous priming.^10–12,14^ In this study we compare MiECC to OpECC which uses all the strategies mentioned.

Currently, the body of literature examining red blood cell usage suggests MiECC confers an advantage over CECC. In a review of 26 randomized control trials (RCTs) comparing MiECC to CECC by Anastasiadis et al., they reported that only 17.5% of patients in the MiECC group required red blood cell transfusion compared to 43.1% of patients that received conventional bypass.^5^ A similar conclusion was drawn by Zangrillo et al. in a systematic review of 16 RCTs, finding that red blood cell transfusion occurred in 9.9 vs 17.9% of cases with MiECC and CECC respectively.^15^ In the present study, hemodilution from initiation to termination of CPB was significantly lower in the MiECC group. Despite superior preservation of functional platelets and hemoglobin in the MiECC group following CPB, no difference was found between groups in regards to red blood cell transfusion. The relatively few number of patients, and lack of randomization, may explain why we did not see a statistical difference in transfusion, despite a trend towards less RBC usage in the MiECT group. Additionally, the BSA of patients in the MiECC group was significantly lower than those who received conventional bypass, thereby increasing the relative amount of hemodilution, potentially increasing risk for RBC transfusion. Furthermore, the priming volume of conventional CPB circuits found in the aforementioned reviews were considerably higher than those achieved in the present study using OpECC. Anastasiadis et al. found the average priming volume of CECC to be over a full liter higher than the MiECC group (p (614 ± 174 vs 1640 ± 158 ml, *p* < 0.001).^5^ Furthermore, both reviews include a study that used 2100 ml to prime their conventional bypass circuit.^16^ A OpECC circuit that aims to minimize priming volume is used at our centre, employing a circuit design strategy that minimizes disposable components and excess tubing length, with a goal to employ retrograde autologous priming in every patient. Approaching conventional CPB in this way closes the priming gap found between CECC and MiECC, potentially contributing to the lack of RBC transfusion differences between groups when compared to the present literature.

In regards to patient outcomes, there were no statistical differences found between the two groups. In a couple of cases a large amount of bleeding from the venous cannulation site was handled effectively with our type III MiECC circuit by temporarily employing the use of pump suction. Air coming down the venous line occurred in a few cases, and was effectively handled with the bubble trap and air purge system. We found that our type III MiECC circuit was able to handle the challenges of CPB, with zero occurrences of having to convert to an open system.

This study is limited by the nature of being a retrospective, non-randomized set of data. Additionally, the groups were not well matched with respect to BSA. Patients in the MiECC group were, on average, smaller than those who received conventional bypass. Furthermore, there may be a learning curve among the team as these MiECC cases represent the initial cohort of patients using this method of CPB.

## Conclusions

Despite our learning curve implementing a MiECC system, and possibly confounded by a smaller average BSA in the MiECC cohort, the use of minimally invasive extracorporeal circulation showed an increased preservation of functional platelets compared to a conventional system optimized for lower prime volumes and biocompatibility. MiECC was not associated with any difference in red blood cell transfusion, or increased mortality. Minimally invasive extracorporeal circulation remains an attractive alternative to optimized conventional cardiopulmonary bypass.
